# Correction

**DOI:** 10.1080/19420862.2024.2416272

**Published:** 2024-10-20

**Article title**: Systematic analysis of Fc mutations designed to reduce binding to Fc-gamma receptors

**Authors**: Geoff Hale, Jelle De Vos, Alastair Douglas Davy, Koen Sandra and Ian Wilkinson

**Journal**: mAbs

**DOI**: https://doi.org/10.1080/19420862.2024.2402701

The author has identified a correction in Table 1 row 6, entry mAb-028: The figure 6 under “Number of INNs” to be replaced by 0 instead of the value 6. The corrected table 1 has been placed below.

Also, in page 6 in the sentence “Besides STR, the only IgG1 variant to completely eliminate FcγR binding was F233Δ/L234Δ/L235Δ” the value F233Δ has been corrected as E233Δ.

The corrected sentence will appear in the article as “Besides STR, the only IgG1 variant to completely eliminate FcγR binding was E233Δ/L234Δ/L235Δ”.

**Table:** 

Sample number	Isotype	Mutations	Number of INNs	IMGT Code	Citation	doi
mAb-024	IgG1		402			
mAb-075	IgG1	E233del L234del L235del	0	G1v65		
mAb-029	IgG1	E233P L234V L235A	1		Armour 1999	10.1002/(SICI)1521-4141(199908)29:08<2613::AID-IMMU2613>3.0.CO;2-J
mAb-030	IgG1	E233P L234V L235A G236del	0	G1v50	Armour 1999	10.1002/(SICI)1521-4141(199908)29:08<2613::AID-IMMU2613>3.0.CO;2-J
mAb-027	IgG1	E233P L234A L235A G236del P329A	2			
mAb-028	IgG1	E233P L234V G236del S267K	0			
mAb-033	IgG1	L234A L235A	28	G1v14	Xu 2000	10.1006/cimm.2000.1617
mAb-034	IgG1	L234A L235A G237A	7	G1v14-1	Bloom 2009	Patent application WO2009143536
mAb-066	IgG1	L234A L235A G237A P238S H268A A330S P331S	0		Tam 2017	10.3390/antib6030012
mAb-036	IgG1	L234A L235A G237A N297A	1			
mAb-035	IgG1	L234A L235A G237A K322A	1		Hernandez-Hoyos 2016	10.1158/1535-7163.MCT-15-024
mAb-061	IgG1	L234A L235A D265A	0			
mAb-056	IgG1	L234A L235A D265S	1	G1v14-67	Edavettal 2022	10.1016/j.medj.2022.09.007
mAb-062	IgG1	L234A L235A K322A	0		Lin 2013	10.4161/cbt.26106
mAb-068	IgG1	L234A L235A L328R	0	G1v14-48		
mAb-057	IgG1	L234A L235A P329A	0	G1v14-4		
mAb-037	IgG1	L234A L235A P329G	10	G1v14-49	Schlothauer 2016	10.1093/protein/gzw040
mAb-058	IgG1	L234A L235A P329S	0			
mAb-038	IgG1	L234A L235A P331S	1	G1v40	Kurnellas 2023	10.1186/s12967-023-04251-y
mAb-039	IgG1	L234A L235E	1			
mAb-040	IgG1	L234A L235E G237A	3	G1v43	Latour 2001	10.4049/jimmunol.167.5.2547
mAb-041	IgG1	L234A L235E G237A A330S P331S	4		Li 2018	10.1186/s40425-018-0329-7
mAb-042	IgG1	L234A L235Q K322Q	1		Alvarado 2022	10.1111/all.15262
mAb-031	IgG1	L234A G237A	1		Bloom 2009	Patent application WO2009143526
mAb-032	IgG1	L234A G237A A330V	1			
mAb-043	IgG1	L234F L235E D265A	3		Engleberts 2020	10.1016/j.ebiom.2019.102625
mAb-044	IgG1	L234F L235E P331S	7	G1v39	Oganesyan 2008	10.1107/S0907444908007877
mAb-064	IgG1	L234F L235Q K322Q	0	G1v53	Borrok 2017	10.1002/eji.1830230216
mAb-067	IgG1	L234S L235T G236R	0	G1v59	Wilkinson 2021	10.1371/journal.pone.0260954
mAb-045	IgG1	L235A G237A	4		Frewin 2002	Patent application WO2002102853
mAb-069	IgG1	L235E	0	G1v23	Alegre 1992	10.4049/jimmunol.148.11.3461
mAb-046	IgG1	L235G G236R	1		Geuijen 2018	10.1016/j.ccell.2018.04.003
mAb-055	IgG1	L235R G236R S239K A327G A330S P331S	1			
mAb-063	IgG1	G236R L328R	0	G1v52	Horton 2008, Chu 2008	10.1158/0008-5472.CAN-08-2268
mAb-074	IgG1	P238S	0	G1v63		
mAb-059	IgG1	D265A	0	G1v66	Shields 2001	10.1074/jbc.M009483200
mAb-026	IgG1	D265A P329A	1			
mAb-060	IgG1	D265S	0	G1v67		
mAb-072	IgG1	S267K	0	G1v51		
mAb-054	IgG1	V273E	1		Ye 2019	10.1158/2326-6066.CIR-18-0805
mAb-047	IgG1	N297A	12	G1v29	Bolt 1993	10.1002/eji.1830230216
mAb-048	IgG1	N297A K322A	1		Hoseini 2018	10.1158/0008-5472.CAN-08-2268
mAb-049	IgG1	N297G	16	G1v30	Leabman 2013	10.4161/mabs.26436
mAb-050	IgG1	N297H	1		Tao 1989	10.4049/jimmunol.143.8.2595
mAb-051	IgG1	N297Q	2	G1v36	Tao 1989	10.4049/jimmunol.143.8.2595
mAb-052	IgG1	N297S	1		He 2013	10.4049/jimmunol.1300409
mAb-065	IgG1	S298N T299A Y300S	0		Zhou 2020	10.1080/19420862.2020.1814583
mAb-053	IgG1	T299A	1		Sazinsky 2008	10.1073/pnas.0809257105
mAb-070	IgG1	L328R	0	G1v48		
mAb-071	IgG1	P329G	0	G1v49	Schlothauer 2016	10.1093/protein/gzw040
mAb-073	IgG1	A330S P331S	0	G1v60		
mAb-025	IgG1	A330V	2		Knopf 2007	Patent application WO2007062188
mAb-001	IgG2		42			
mAb-008	IgG2	V234A G237A	2		Cole 1997	10.4049/jimmunol.159.7.3613
mAb-009	IgG2	V234A G237A P238S H268A V309L A330S P331S	1	G2v3	Vafa 2014	10.1016/j.ymeth.2013.06.035
mAb-011	IgG2	V234S A235T del236R	0		Wilkinson 2021	10.1371/journal.pone.0260954
mAb-005	IgG2	D265A A330S P331S	1		Bloom 2009	Patent application WO2009143526
mAb-076	IgG2	H268Q V309L A330S P331S	0	G2v2	An 2009	10.1111/all.15262
mAb-006	IgG2	F296A N297Q	0			
mAb-007	IgG2	K322A	1			
mAb-004	IgG2	A330S P331S	5		Armour 1999	10.1002/(SICI)1521-4141(199908)29:08<2613::AID-IMMU2613>3.0.CO;2-J
mAb-010	IgG2	P331S	2		Yan 2011	Patent application W2011147319
mAb-002	IgG2/1	F296A N297Q	1		Goodman 2009	Patent application WO2009010290
mAb-003	IgG2/4		4	G2G4v1	Mueller 1997	10.1016/S0161-5890(97)00042-4
mAb-022	IgG3		1			
mAb-023	IgG3	L234S L235T G236R	0		Wilkinson 2021	10.1371/journal.pone.0260954
mAb-012	IgG4		29		Bruggemann 1987	10.1084/jem.166.5.1351
mAb-013	IgG4-P		95		Angal 1993	10.1016/0161-5890(93)90432-B
mAb-018	IgG4-P	E233P F234V L235A G236del	4		Armour 1999	10.1002/(SICI)1521-4141(199908)29:08<2613::AID-IMMU2613>3.0.CO;2-J
mAb-017	IgG4-P	E233P F234V L235A D265A	1		Zhang 2018	10.1007/s00262-018-2160-x
mAb-014	IgG4-P	F234A L235A	17	G4v4	Alegre 1994	10.1097/00007890-199457110-00001
mAb-020	IgG4-P	F234A L235A G237A P238S	0		Tam 2017	10.3390/antib6030012
mAb-021	IgG4-P	F234S L235T G236R	0		Wilkinson 2021	10.1371/journal.pone.0260954
mAb-015	IgG4-P	L235A	1		Smith 2008	Patent application WO2008129124
mAb-016	IgG4-P	L235E	8	G4v3	Alegre 1992	10.4049/jimmunol.148.11.3461
mAb-077	IgG4-P	L235E P329G	0	G4v3-49	Schlothauer 2016	10.1093/protein/gzw040
mAb-019	IgG4-P	N297Q	1	G4v36	Schlothauer 2016	10.1093/protein/gzw040

